# Second-Generation SARS-CoV-2 Recombinants: Lessons from Other Viruses

**DOI:** 10.3390/v15051063

**Published:** 2023-04-26

**Authors:** Daniele Focosi, Fabrizio Maggi

**Affiliations:** 1 North-Western Tuscany Blood Bank, Pisa University Hospital, 56124 Pisa, Italy; 2 National Institute for Infectious Diseases “Lazzaro Spallanzani”, 00149 Rome, Italy; fabrizio.maggi@inmi.it

RNA viruses have developed notable strategies to evolve and escape host immunity. For non-segmented viruses, which represent most viral species, cumulative mutations (antigenic drift) are the main way of evolution. However, when two different RNA viruses infect the same cell, they can undergo two forms of recombination, both leading to antigenic shifts: RNA recombination and reassortment, with the latter restricted to viruses with segmented genomes [1]. For example, homologous recombination plays only a very minor role in the evolution of human influenzavirus A [2], which instead can reassort the RNA segments of its genome (intra- or inter-subtypic reassortment), as well as undergo intragenic recombination between different RNA segments (commonly referred to as nonhomologous recombination [3,4,5]), as well as intragenic recombination between viral RNA and exogenous RNA [6].

Despite being theoretically possible in any RNA virus, the rates of recombination vary markedly among RNA viruses. Some viruses, particularly those with negative-sense single-stranded genomes, exhibit such low rates of recombination that they are effectively clonal. Although recombination is often represented as a form of sexual reproduction, there is little evidence that recombination in RNA viruses evolved as a way of creating advantageous genotypes or removing deleterious mutations. In particular, there is no association between recombination frequency and the burden of a deleterious mutation. Similarly, there is little evidence that recombination could have been selected as a form of genetic repair.

Some retroviruses such as HIV recombine rapidly because their virions contain two genome copies and template switching between these copies is unavoidable during the viral replication cycle. Recombination in RNA viruses is hence likely a mechanistic by-product of the processivity of the viral polymerase that is used in replication, and it varies with genome structure. Negative-sense single-stranded RNA viruses may recombine at low rates because of the restrictive association of genomic RNA in a ribonucleoprotein complex, as well as a lack of substrates for template switching. Some positive-sense single-stranded RNA viruses exhibit high rates of recombination that can exceed the rates of single nucleotide variations. Comparison across multiple viruses suggests an inverse correlation between genome length and recombination rate. Patino-Galindo et al. detected high levels of recombination in Sarbecoviruses, HBV, HEV, Rhinovirus A, and HIV. Noteworthy recombination hotspots are found in MERS-CoV and Sarbecoviruses (at Spike, Nucleocapsid and ORF8) [7]. Of interest, recombination has also been recently reported for DNA viruses, in particular, Anelloviridae [8].

Second-generation recombinants are defined as recombinants of (first-generation) recombinant lineages. To have second-generation recombinants, several conditions are needed:

- Different sublineages should exist, including a growing share of first-generation recombinants;
- Different sublineages should circulate at high levels (syndemic) to facilitate co-infection in different lineages;
- High levels of viral surveillance (genome sequencing), making detection and tree reconstruction feasible.

Second-generation recombinants have been so far documented for several viruses:

- Retroviruses:
  ○ HIV-1: at present, 106 circulating recombinant forms (CRFs) have been identified worldwide, with many more unique recombinant forms (URF). URFs are responsible for at least 20% of HIV-1 infections worldwide—a proportion that reaches 40% in some African countries [9]. Inter-subtype recombinant viruses, especially CRF01_AE and CRF07_BC, have become the predominant strains of the HIV-1 epidemic in China [10]. The double infection between CRFs established more optimal conditions for the second-generation recombination, and many second-generation recombinants were represented by the recombination of CRF01_AE and CRF07_BC fragments, such as CRF79_0107 [11], CRF80_0107 [12], CRF102_01075 [13], CRF109_0107 [14], CR125_0107 [15], and others [16,17,18,19,20,21,22,23,24]. Other instances include CRF01_AE x CRF08_BC [25], CRF01_AE x subtype B [26], CRF07_BC x CRF83_cpx [27], and CRF86_BC x a URF [28] also from in China.
  ○ HTLV [29].
- HBV X/C [30] and complex A/C/G recombinants [31].
- ssRNA viruses.
  ○ Enterovirus [29].
  ○ Alphacoronavirus: Clade B (Serotype II) Feline coronavirus (Alphacoronavirus 1) [32].
  ○ Betacoronavirus:
    ▪ SARS-CoV-2 [33,34]: when it comes to SARS-CoV-2, the nomenclature of recombinants is managed so far only by the PANGOLIN phylogeny, which ante poses the “X” prefix. With 2372 sublineages designated as of 14 April 2023 (including 920 labeled as Omicron by the WHO), there have been 58 recombinant lineages (excluding designated progenies). The diversification of progenies has led to fourth-level descendants, which, according to PANGOLIN rules, should be shortened using aliases: the point here is that rules impose the next available alias without the X prefix, preventing the immediate understanding of whether the originating lineage was or not a recombinant [35].
- XBL is an XBB.1* where part of ORF1a has been replaced with BA.2.75.* [36].
- XBY is XBF x BR.2.1.
- XBW is XBB.1.5 x BQ.1.14.
- XBV is XBB.1 x CR.1.

In conclusion, while reconstructing, further-generation recombination might sound like a mere style exercise, but it has huge implications for vaccine preparedness and seroepidemiology, recombination being a key driver of immune escape. With widespread endemic circulation, more and more second- and further-generation SARS-CoV-2 recombinant lineages are expected, with some of them posing a theoretical concern for increased disease severity and vaccine efficacy.

## References

[1] Simon-Loriere E., Holmes E.C. (2011). Why do RNA viruses recombine?. Nat. Rev. Microbiol..

[2] Boni M.F., Zhou Y., Taubenberger J.K., Holmes E.C. (2008). Homologous Recombination Is Very Rare or Absent in Human Influenza A Virus. J. Virol..

[3] Bergmann M., García-Sastre A., Palese P. (1992). Transfection-mediated recombination of influenza A virus. J. Virol..

[4] Orlich M., Gottwald H., Rott R. (1994). Nonhomologous recombination between the hemagglutinin gene and the nucleoprotein gene of an influenza virus. Virology.

[5] Suarez D.L., Senne D.A., Banks J., Brown I.H., Essen S.C., Lee C.W., Manvell R.J., Mathieu-Benson C., Moreno V., Pedersen J.C. (2004). Recombination resulting in virulence shift in avian influenza outbreak, Chile. Emerg. Infect. Dis..

[6] Khatchikian D., Orlich M., Rott R. (1989). Increased viral pathogenicity after insertion of a 28S ribosomal RNA sequence into the haemagglutinin gene of an influenza virus. Nature.

[7] Patiño-Galindo J., Filip I., Rabadan R. (2021). Global Patterns of Recombination across Human Viruses. Mol. Biol. Evol..

[8] Arze C.A., Springer S., Dudas G., Patel S., Bhattacharyya A., Swaminathan H., Brugnara C., Delagrave S., Ong T., Kahvejian A. (2021). Global genome analysis reveals a vast and dynamic anellovirus landscape within the human virome. Cell Host Microbe.

[9] Hemelaar J., Gouws E., Ghys P.D., Osmanov S. (2011). Global trends in molecular epidemiology of HIV-1 during 2000–2007. AIDS.

[10] Li X., Li W., Zhong P., Fang K., Zhu K., Musa T.H., Song Y., Du G., Gao R., Guo Y. (2016). Nationwide Trends in Molecular Epidemiology of HIV-1 in China. AIDS Res. Hum. Retrovir..

[11] Li Y., Feng Y., Li F., Xue Z., Hu J., Xing H., Ruan Y., Shao Y. (2017). Genome Sequence of a Novel HIV-1 Circulating Recombinant Form (CRF79_0107) Identified from Shanxi, China. AIDS Res. Hum. Retrovir..

[12] Zhang Y., Pei Z., Li H., Han J., Li T., Li J., Liu Y., Li L. (2019). Characterization of a Novel HIV-1 Circulating Recombinant Form (CRF80_0107) Among Men Who Have Sex with Men in China. AIDS Res. Hum. Retrovir..

[13] Li X., Wu J., Zhang Y., Shen Y., Li H., Xing H., Liu Y., Yang X., Ding X., Hu B. (2019). Characterization of a novel HIV-1 second-generation circulating recombinant form (CRF102_0107) among men who have sex with men in Anhui, China. J. Infect..

[14] Wang X., Zhao J., Li X., Li H., Zhang Y., Liu Y., Chen L., Zheng C., Jia L., Han J. (2020). Identification of a novel HIV-1 second-generation Circulating Recombinant Form CRF109_0107 in China. J. Infect..

[15] Xiao M., Feng Y., Gao L., Yang C., Liu J., He M., Li J., Zhang M., Dong X., Xia X. (2022). Characterization of a Newly Emerging HIV-1 Second-Generation Recombinant Form (CRF125_0107) Among Heterosexuals in Yunnan, China. J. Infect..

[16] Jiang J., Liang B., Li K., Yang Y., Yang Y., Ning C., Zhang F., Wei Q., Liang H., Ye L. (2020). Genomic Characterization of a Novel HIV Type 1 Strain Originating from CRF07_BC and CRF01_AE by Heterosexual Transmission in the Lingshan Prefecture of Guangxi Province, China. AIDS Res. Hum. Retrovir..

[17] Xing Y., Guo Y., Wang L., Li H., Han J., Wang X., Liu Y., Jia L., Li J., Bai H. (2021). Identification of Two Novel HIV-1 Second-Generation Recombinant Forms (CRF01_AE/CRF07_BC) in Hebei, China. AIDS Res. Hum. Retrovir..

[18] Fan W., Liu Y., Li Y., Su M., Meng J., Lu X., Shi P. (2022). Identification of Three Novel HIV-1 Second-Generation Recombinant Forms (CRF01_AE/CRF07_BC) Among Men Who Have Sex with Men in Baoding, Hebei, China. AIDS Res. Hum. Retrovir..

[19] Yin Y., Zhou Y., Lu J., Guo H., Chen J., Xuan Y., Cheng H., Yuan D., Hu H., Xu X. (2021). The Identification of A Novel HIV-1 Second-Generation Recombinant form (CRF01_AE/CRF07_BC) Among Men Who Have Sex with Men in Jiangsu, China. Curr. HIV Res..

[20] Yin Y., Zhou Y., Lu J., Guo H., Chen J., Xuan Y., Yuan D., Hu H., Xu X., Fu G. (2021). First Detection of a Cluster Novel HIV-1 Second-Generation Recombinant (CRF01_AE/CRF07_BC) among Men Who Have Sex with Men in Nanjing, Eastern China. Intervirology.

[21] Zhu B., Zhao J., Wang X., Li H., Liu Y., Zheng C., Jia L., Li T., Wang X., Chen L. (2022). Characterization of Three Novel HIV-1 Second-Generation Recombinants (CRF01_AE/CRF07_BC) Identified in Shenzhen, China. AIDS Res. Hum. Retrovir..

[22] Cheng S.W., Chen Y.P., Qi F., Gao R., Ma K.L., Yang P., Xie Y.N., Gao L.L., Chen X., Zhou Y.H. (2021). Genetic Characterization of a Novel HIV-1 Second-Generation Recombinant Form (CRF01_AE/CRF07_BC) Identified in Yan’an City, China. AIDS Res. Hum. Retrovir..

[23] He S., Gao Y., An M., Zhao B., Wang L., Ding H., Han X. (2021). Characterization of a Novel HIV-1 CRF01_AE/CRF07_BC Recombinant Strain Among Men Who Have Sex with Men in Liaoning, China. AIDS Res. Hum. Retrovir..

[24] Lu X., Zhang X., An N., Tang X., Wang Y., Liu M., Yan L., Zhang Y., Li Q. (2023). Recombinant characteristics of three novel HIV-1 second-generation recombinant forms composed of CRF01_AE and CRF07_BC isolated in Hebei province, China. Arch. Virol..

[25] Li J., Li L., Li H., Li J., Yang S., Zhang M., Ouyang H. (2016). Genomic Characterization of a Novel HIV-1 Second-Generation Recombinant Form Originated from CRF01_AE and CRF08_BC in Dali Prefecture of Yunnan Province, China. AIDS Res. Hum. Retrovir..

[26] Tang X., Liu M., An N., Zhang X., Wang Y., Li Y., Li Q., Lu X. (2023). Identification of Two Novel HIV-1 Recombinant Forms Originated from CRF01_AE and Subtype B in the Sexual Contact Population in Hebei Province, China. AIDS Res. Hum. Retrovir..

[27] Xie Y.N., Li S.L., Yang R.R., Huang J., Peng X., Xu W., Cheng S.W., Zhou Y.H., Chen X., Li H. (2021). Genetic Characteristics of Three Unique Recombinant Forms of HIV-1 in Yunnan, China. AIDS Res. Hum. Retrovir..

[28] Chen Y., Li Z., Xie Y., Zhao L., Hu A., An L., Dong X., Liu D., Ma Q., Chen X. (2021). Genetic Characterization of a Novel HIV-1 Second-Generation Recombinant Form Originating from CRF86_BC and a Unique Recombinant Form in Yunnan, China. AIDS Res. Hum. Retrovir..

[29] Nikolaidis M., Mimouli K., Kyriakopoulou Z., Tsimpidis M., Tsakogiannis D., Markoulatos P., Amoutzias G.D. (2019). Large-scale genomic analysis reveals recurrent patterns of intertypic recombination in human enteroviruses. Virology.

[30] Fang Z.L., Hué S., Sabin C.A., Li G.J., Yang J.Y., Chen Q.Y., Fang K.X., Huang J., Wang X.Y., Harrison T.J. (2011). A complex hepatitis B virus (X/C) recombinant is common in Long An county, Guangxi and may have originated in southern China. J. Gen. Virol..

[31] Su H., Liu Y., Xu Z., Cheng S., Ye H., Xu Q., Liu Q., Tan S., Xu D., Liu Y. (2014). A novel complex A/C/G intergenotypic recombinant of hepatitis B virus isolated in southern China. PLoS ONE.

[32] Jaimes J.A., Millet J.K., Stout A.E., André N.M., Whittaker G.R. (2020). A Tale of Two Viruses: The Distinct Spike Glycoproteins of Feline Coronaviruses. Viruses.

[33] Focosi D., Maggi F. (2022). Recombination in Coronaviruses, with a focus on SARS-CoV-2. Viruses.

[34] Markov P.V., Ghafari M., Beer M., Lythgoe K., Simmonds P., Stilianakis N.I., Katzourakis A. (2023). The evolution of SARS-CoV-2. Nat. Rev. Microbiol..

[35] Focosi D., Maggi F. (2023). How SARS-CoV-2 Big Data Are Challenging Viral Taxonomy Rules. Viruses.

[36] XBB.1*/BA.2.75*/XBB.1* Recombinant with S:F486P (8 seq, Malaysia) #1532. https://github.com/cov-lineages/pango-designation/issues/1532.

